# And the nominees are: Using design-awards datasets to build computational aesthetic evaluation model

**DOI:** 10.1371/journal.pone.0227754

**Published:** 2020-01-21

**Authors:** Baixi Xing, Kejun Zhang, Lekai Zhang, Xinda Wu, Huahao Si, Hui Zhang, Kaili Zhu, Shouqian Sun

**Affiliations:** 1 Institute of Industrial Design, Zhejiang University of Technology, Hangzhou, China; 2 College of Computer Science and Technology, Zhejiang University, Hangzhou, China; 3 School of Media and Design, Hangzhou Dianzi University, Hangzhou, China; Nanjing University of Information Science and Technology, CHINA

## Abstract

Aesthetic perception is a human instinct that is responsive to multimedia stimuli. Giving computers the ability to assess human sensory and perceptual experience of aesthetics is a well-recognized need for the intelligent design industry and multimedia intelligence study. In this work, we constructed a novel database for the aesthetic evaluation of design, using 2,918 images collected from the archives of two major design awards, and we also present a method of aesthetic evaluation that uses machine learning algorithms. Reviewers’ ratings of the design works are set as the ground-truth annotations for the dataset. Furthermore, multiple image features are extracted and fused. The experimental results demonstrate the validity of the proposed approach. Primary screening using aesthetic computing can be an intelligent assistant for various design evaluations and can reduce misjudgment in art and design review due to visual aesthetic fatigue after a long period of viewing. The study of computational aesthetic evaluation can provide positive effect on the efficiency of design review, and it is of great significance to aesthetic recognition exploration and applications development.

## Introduction

Computer-aided design evaluation is becoming a well-recognized request in the intelligent design industry. Such a evaluation tool can be used as an intelligent assistant to human assessors, helping reduce misjudgments due to visual aesthetic fatigue and efficiently performing the manual work of primary screening. Vast amounts of design concepts have been created and nurtured for submission to various design awards competitions. The archives of original design submissions are rich potential data resource for aesthetic-aware modeling.

Design is an interdisciplinary major, combining engineering and art. Using visual and affective perception, human beings establish the direct image of design works, while on the other hand, layouts following design principles reveal certain forms created by rational thinking. Various factors should be considered in the concept creation process, including human factors, ergonomics, environment, psychology, and safety etc [1, 2]. Thus, excellent design layout is the creation of both art and engineering, and this combination of patterns is quite challenging for computer-based assessment study. Among all the factors, visual aesthetics is proved to be critical in product design evaluation [3, 4] by physiological analysis approaches [5] and user experience study [6]. The existing studies indicated that visual aesthetics is significantly influencing user preference [7] and stimulating users’ purchase behavior [8–10], which has a crucial effect on promoting product acceptance [11]. Consequently, quantifying the visual aesthetics of design works by computational means is promising for various industries.

In this study, we explore an aesthetic-aware model of design assessment using image-feature analysis. The main contributions of this work include the following content. A total of 2,918 images of original design works were collected from archived submissions to two industrial design awards to form two databases for design evaluation, and multiple machine-learning methods were compared to find the optimal method for automatic design grading. Specifically, the ranking information and reviewers’ ratings are the natural classification annotations of these design images. In this experiment, the following image features were extracted as hand-crafted features for aesthetic modeling by LibSVM, LibLinear, RBFNetwork and RandomSubSpace-Randomforest: local binary pattern (LBP), color histogram (HIST), and hue saturation value (HSV). VGG-19 and ResNet-50 were also used in the design aesthetic classification learning. The experimental results were assessed as optimal, attaining a classification accuracy of 80.19% on average, through the applying use of ResNet-50 in the dataset of submissions to the Electronic Home Applicants Design Awards. The methodology was then verified with the use of a second dataset, taken from submissions to the Electronic Tools Design Awards. The modeling performance was found to be stable, with an accuracy of 84.19% in selecting the nominees.

This paper is organized as follows: Section 2 summarizes the previous work on aesthetic evaluation using image processing methods; Section 3 introduces the methods of feature extraction and algorithms, including LibSVM, LibLinear, RBFNetwork, RandomSubSpace, VGG-19 and ResNet-50; Section 4 gives the experimental procedures, including reviewers’ evaluations based on design criteria and the design works evaluation using multi-modal modeling of image features; Section 5 presents the results and discusses the experiments; and Section 6 provides the conclusion of the study and directions for future study.

## Related works

The joint study of aesthetic factors in art and design is of great importance for studies of multimedia computing, intelligent design, and aesthetics culture. In addition, aesthetic values in art and design are unique features of in cultural and social development, which develops various artistic forms over time. This can be an extremely important evidence for the study of features of visual perception. Thus, the aesthetic principles and patterns of multimedia works should be explored using computer models [12–15]. Here we review the related works of multimedia aesthetic computing, and the existing multimedia aesthetic databases are concluded.

### 2.1 Multimedia aesthetic modeling works

A review of aesthetic-aware modeling research is given in Table 1. Works that attempt to bridge computing and the perception of aesthetics have three main tasks. First, aesthetic images are to be evaluated using qualitative measures [16–18], including user interface design [19–21], photos, paintings, and filmed scenes. Second, multimedia retrieval is to be developed, based on aesthetic recognition. Third, aesthetic multimedia is to be generated using aesthetic-aware modeling [22, 23]. Work in this area can produce various applications, such as in the intelligent assistance of design evaluation and of design of computer games [24].

**Table 1:** Multimedia aesthetic-aware modeling approaches.

Refer	Features	Classifier/Method	Descriptors	Dataset	Annotators	Results
Tian, 2018 [13]	Image	DCNNs	Aesthetic ranking	AVA	N/A	Kendall’s tau-b: 0.487
Liao, 2014 [14]	Image Visual elements	Statistics	Balance Complexity Repetition	26000 Logos	N/A	Output scores: Balance Complexity Repetition
Sheng, 2018 [15]	Image	Multi-patch aggregation method	Aesthetic Positive/ Negative	AVA	N/A	Catalyst: 83.03%
Qian, 2018 [16]	Image (SIFT, RGB)	Crowdsourced saliency map	Image Summarization	POI images 7 millions	7387 Users	POI summarization
Ren, 2017 [17]	Image	Active learning algorithm	Aesthetic score	FLICKR- AES	Amazon Mechanical Turk	Direct score prediction: 0.039
Kucer, 2018 [18]	Hand-crafted features Meta features	VGG-16 VGG-19 ResNet-50	Mean aesthetic Score High/Low	HB AVA	N/A	Accuracy: 81.95%
Chen, 2016 [19]	Image (color, structure, complexity, texture)	Fuzzy-rule-based method	Aesthetic rating	Webpage dataset	N/A	Better predictive ability than linear regression model
Maity, 2019 [20]	Image, text, white space	SVM	Aesthetic	250 images 95 text samples	83 Users/ 185 Users	Image RMSE: 0.68 Text RMSE: 0.56
Persada, 2017 [21]	Information architecture Navigation design User interface	Kansei engineering Factor analysis	Feasibility	20 websites	47 Students	Feasibility of use
Wu, 2016 [22]	Pattern Image	Cloud model	N/A	N/A	N/A	Pattern generation
Zhang, 2018 [23]	Image (color, saturation, contrast, brightness, others)	Stack GAN Bimodal Deep Autoencoder	Aesthetic	4000 Paintings	10 Students	Image generation
Erdem, 2016 [24]	Image (composition, texture, line)	LSBoost BAG RF	High/Low	AVA	N/A	MSE: 0.394
Brain, 2006 [25]	Image	Ralph’s model of aesthetics	Score of deviation from normality	Images of Fine Art	N/A	Visually harmonious image synthesis
Wong, 2009 [26]	Image	SVM	High/Low	3161 photos	N/A	Accuracy: 78.8%
Su, 2012 [27]	Image (color, texture, saliency map, etc.)	Adaboost	High/Low	DP Challege	N/A	Accuracy: 92.06%
Lovato, 2014 [28]	Image (HSV, entropy, wavelet, textures, etc.)	LASSO Regressor	High/Low	Flickr Images	200 Individuals	Accuracy: 96%
Zhang, 2014 [29]	Visual graphic features	Embedded algorithm	High/Low	AVA CUHK PNE	N/A	Probabilistic: AVA: 83.24% CUHK: 90.31% PNE: 83.02%
Tarvainen, 2014 [30]	Visual Auditory Temporal	Extreme Learning Machine	Aesthetic	Movie clips	73 Viewers	Prediction deviation ratio: 1.19
Temel, 2014 [31]	Image (SIFT, CN, DOG, DOSA)	GIST, GMM, DOG	Aesthetic score	AVA	N/A	SIFT: 75.5% CN: 74.0% DOG: 72.6% DOSA: 69.7%
Wu, 2016 [32]	Image (structural, local, global visual features)	SVM, SSVM, BPNN	Aesthetic	Webpage	N/A	Testing Errors: SVM:0.7716 SSVM: 0.7605 BPNN:0.8004
Lu, 2015 [33]	Image (global, local image features)	RDCNN	Aesthetic	AVA IAD	N/A	Accuracy:75.41%
Jin, 2016 [34]	Image	DCNNs	Low/High	AVA	N/A	MSE: 97.54%
Lee, 2017 [35]	Image	DCNNs	Low/High	AVA	N/A	Accuracy:81.02%
Liu, 2019 [36]	Deep GSP image features	SDAL	Human gaze shifting path consistency	Millions Flickr Photos	200,000Users	Consistency: 93%
Wang, 2017 [37]	Image	DNN	Low/High	AVA MSR-ICD	N/A	AVA- Accuracy:76.9% MSR-ICD -BDE: 0.032
Tong, 2017 [38]	Geometrical appearance features	DCNNs	Pleasant/ Unpleasant	4240 faces	N/A	Proved that pleasant faces matched the golden ratio proportions
Fu, 2018 [39]	Image (global, local, scene)	DCNNs(VGG-16, ResNet-50)	Low/High	AVA CUHKPQ	N/A	Accuracy: 90.01%
Murray, 2012 [42]	Image (SIFT, LBP, color)	SVMs	60 categories Low/High	AVA	Hundreds of amateur and professional photographers	mAP: 53.85%
Meng, 2018 [43]	Image	MobileNet, VGG, Inception-v3	Aesthetic score	AVA	N/A	Accuracy: 79.38%
Sidhu, 2018 [44]	Image(HSV,RGB, Entropy,brightness, etc.)	Regression Models	Beauty/liking ratings	480 paintings	598 undergraduates	Adjusted R^2^: 0.13

(LSBoost: Least-squares Boosting; BAG: Bagged Tree Ensembles; RF: Random Forests; POI: Place of Interest; SDAL: Semi-supervised Deep Active Learning; DNN: Deep Neural Networks; CNN: Convolutional Neural Networks; BDE: Boundary Displacement Error; mAP: mean Average Precision, Adjusted R^2^: Adjusted R-square)

There are some specific guiding principles and aesthetic standards found in design theories, involving the treatment of colors and hues, saturation values, and layout formats. It is easy to select features of images that represent such characters. During the early period of aesthetic modeling study, evolutionary methods were commonly used. Ross et al. created an automatic synthesis of aesthetically pleasing images via genetic programming for the generation of textures [25]. Wong et al. presented a saliency-enhanced method for distinguishing professional photographs from amateur ones. A set of salient features and global features were utilized in this study [26]. Su et al. proposed a preference-aware image aesthetic model, which covered both implicit and explicit aesthetic features to meet users’ preferences, and the model achieved an accuracy rate of 92.06%. They also found that contrast features were most effective among the tested information [27]. Lovato et al. developed a personal aesthetic model as a novel behavioral biometrical trait, assessing low- and high-level features of Flickr images, using a LASSO (Least absolute shrinkage and selection operator) regressor [28]. Zhang et al. evaluated aesthetic quality in photographs, encoding local and global structural features [29]. Tarvainen et al. built a film dataset to develop assessments of style, aesthetics, and affect in films. Neural-network based Extreme Learning Machine was experimentally found to be slightly better than linear regression [30]. Temel et al. performed a comparative study of computational aesthetics and found that the feature of generic or hand-crafted was insufficient for aesthetics modeling, and the relationship between features and aesthetics was then explored through deep learning in the further study [31].

Various methods have proven to be useful in aesthetic learning, including SVM [32], GMM, Bayes, DCNNs [33–39], etc. Different images can be distinguished by style, and they often follow different aesthetic rules. Therefore, the best learning approach should differ according to the given dataset [40, 41]. The classic aesthetic database AVA, which contains over 250,000 images and a large amount of meta-data with aesthetic scores, has been used in many studies to produce modeling comparison and optimization [42]. Lu et al. investigated the effectiveness of deep neural networks on a 1.5-million image dataset for aesthetic assessment, finding an accuracy of 75.41% [33]. Using the AVA database, Jin et al. adopted DCNNs for prediction of image aesthetics, which achieved a high performance of means square error 0.3373 [34]. Meng et al. constructed a multi-layer aggregation network with various baseline networks from MobileNet, VGG-16, and Inception-v3. The experimental results indicated that the developed model exhibited superior performance to those found in existing studies [43]. Sidhu et al. explored aesthetic prediction on both beauty ratings and liking ratings for 240 abstract and 240 representational paintings in the study based on regression models. They used 4 subjective and 11objective predictors in the measurement and found that the results varied widely in modeling between abstract and representational paintings [44].

### 2.2 Multimedia aesthetic databases

Multimedia databases for aesthetic evaluation were constructed in various studies. The largest aesthetic database is AVA database (Aesthetic Visual Analysis) [42], which is a widely used aesthetic database containing 250,000 images in 60 classes. The other aesthetic databases include FLICKR-AES [17], HB [18], CUHK [29], PNE [29], MSR-ICD [37] and CUHKPQ [39].

In conclusion, machine learning algorithms have been widely used in aesthetic modeling in recent years for large image datasets, although an unexplored problem remains, namely, that of specific aesthetic learning regarding the styles of artworks and understanding user preferences. The selection of features for different images and differences in approaches to them should receive further exploration in a way that takes into account the goal of assessment.

## Methodologies

In the existing aesthetic computing research, there are three main types of studies, the aesthetic ranking analysis [13], classification of aesthetic level (low/high or positive/negative) [15, 18, 24, 26–29] and the aesthetic score prediction [17, 31, 43]. In the majority of the related works, researchers conducted classification method on image aesthetic computing study. Thus, here we transformed the aesthetic computing problem into a three-class-classification problem of image aesthetic level. The contribution of this work is building a relatively objective aesthetic database with design awards submissions, which is a suitable carrier for aesthetic computing study. And an aesthetic evaluation model for product design is explored based on these datasets.

The work of aesthetic-aware modeling taken from design competition datasets can be considered a classification task. In this study, we built classification models using 10-fold cross-validation. Five algorithms (LibSVM, Liblinear, RBFNetwork, RandomSubSpace, VGG-19 and ResNet-50) were implemented for the three classification divisions, namely, “eliminated”, “middle class”, and “nominees”. The image features extraction approach and the applied methods are introduced below.

### 3.1 Image pre-processing

In the design work submission session, designers were requested to submit the proposal layout in an image format of 300 dpi. In the experiment, the input design layout images were resized to 640 × 480 without any cropping.

There are two original design layouts collections applied in this experiment, one is of 2216 pieces of design works collected from “Electronic Home Applicants Design Award” in 2012, while another collection has 639 submissions from “Electronic Tools Design Award” in 2015. We obtained permission to use the design layout images of the design awards from the awards organizers for this design aesthetic computing research. The images have been collected and classified by the design awards organizer during the design competition.

### 3.2 Feature extraction

Based on these image collections, two kinds of design aesthetics database were built in this study, including the databases of hand-crafted image features and the databases of deep-learning features extracted by deep learning approaches.

(1)Hand-crafted features database

In the hand-crafted databases, the image features of LBP [42] and color features (HSV and HIST) [19, 23, 27, 28, 44]were utilized for the modeling, in view of the previous studies on image aesthetics computing (see Session 2). On the other hand, the selection of features also took into account the industrial design theory of “Comprehensive Formation”, which is the general name of plane formation, color formation and three dimensional composition, the theoretical basis of industrial design. Inspired by the basic design theories, in this aesthetic evaluation study, we extracted the related image feature sets of LBP, HSV and HIST, that can represent contour features and color features of design work. Specifically, 64 Dimensions of LBP, 256 dimensions of HSV and 256 dimensions of HIST were extracted by OpenCV to form the databases.

(2)ResNet-50 features database

The deep-learning features databases were formed by image features extracted by ResNet-50. Firstly, we use ResNet-50 to extract a total of 25,088 dimensions of image features. A total of 2,048 features were obtained as the output vector. Then, it was reduced to be a 512-dimentional features vector by a fully connected network as the neural network input for the next step.

(3)VGG-19 features database

Firstly, a total of 25088 dimensions of image features were extracted by VGG-19. Then, 1000 features were obtained as the output vector. Then, it was reduced to be a 512-dimentional features vector by a fully connected network as the neural network input for the next step.

### 3.3 Algorithms

#### LibSVM

LibSVM is an integrated tool used in multi-class classification and regression. A support vector machine (SVM) is a generalized linear classifier that uses binary classification, with a supervised learning method. Its decision boundary is the maximum-margin hyperplane for learning data samples. SVM uses hinge loss to compute empirical risk. It is regularized in the solution process to optimize its structure. A series of improved and extended algorithms have been developed for this, including multi-class classification, least-square SVM, support vector regression, support vector clustering, and semi-supervised SVM. This approach can be combined with other algorithms to optimize attributes to form various ensemble learning methods. SVM is widely used in pattern recognition and multimedia classification. It has been shown in many studies that this approach is highly efficient for the classification of small datasets.

#### Liblinear

Liblinear is a linear classifier that is suitable for use in the classification of large datasets and multidimensional attributes [45]. It contains multiple classifiers for linear regression and SVM, including L2-regularized classifiers, L2-loss linear SVM, L1-loss linear SVM, and logistic regression (LR), L1-regularized classifiers, L2-loss linear SVM and logistic regression (LR), L2-regularized support vector regression, and L2-loss linear SVR and L1-loss linear SVR. For a sample of the form (*x*_*i*_, *y*_*i*_), *i* = 1,…,*k*, *x*_*i*_∈*D*^*n*^, *y*_*i*_∈{−1,1}, this algorithm can solve problems of unconstrained optimization, as follows:

$$\underset{\alpha }{\min}\frac{1}{2}\alpha^{T}\alpha +\delta \sum_{i=1}^{k}\beta (\alpha ;x_{i},y_{i})$$ (1)

in which *δ*>0 is set as the penalty parameter, while *β*(*α*;*x*_*i*_,*y*_*i*_) represents the loss function. Previous work has shown the advantages of this approach in the classification of large datasets [44].

#### RBFNetwork

A radial basis function network (RBFNetwork) is a neural network that uses a radial basis function for activation. It is usually constructed with three layers: an input layer, a hidden layer, and an output layer. The hidden layer can be represented by *θ*_*i*_:*V*_*n*_→*V*. The output of RBFNetwork is a scalar function of input vectors. This method is widely used to solve problems of function approximation, predication, classification, and regression. With this approach, complex, dimensional input data can be reduced and mapped into a new space. The kernel parameter is optimized with an estimation method. The resulting network output is formed of a combination of the radial basis functions of input data and neuron parameters [46].

#### RandomSubSpace

RandomSubSpace is an ensemble learning method that can combine algorithms for classifiers. It constructs a classifier based on a decision tree and adapts to the highest performance on training dataset, improving its generalization accuracy as it grows in complexity. This algorithm incorporates multiple trees, which are constructed systematically by randomly selecting subsets from feature vectors. The trees are constructed in randomly chosen subspaces. For this situation, it is feasible that the number of features would be much larger than the number of samples, such as datasets of gene sequences and fMRI [47].

#### VGG-19

VGG-19 can achieve great accuracy in the large-scale image recognition [48] and it is also applicable in a number of image aesthetic computing studies [18][39][43]. It is using architecture with small convolution filters. However, a significant result improvement can be achieved by constructing 16–19 weight layers. The VGG-19 network training process is presented as follows:

- Firstly, a fix-size 224 x 224 image is set to be the input during training.
- Secondly, a total of 1,000 dimensional features were extracted by VGG-19.
- Thirdly, the dimensions of image features vector were reduced to be a 512-dimentional features vector by the fully connected network for the classification.

**Fig 1:**
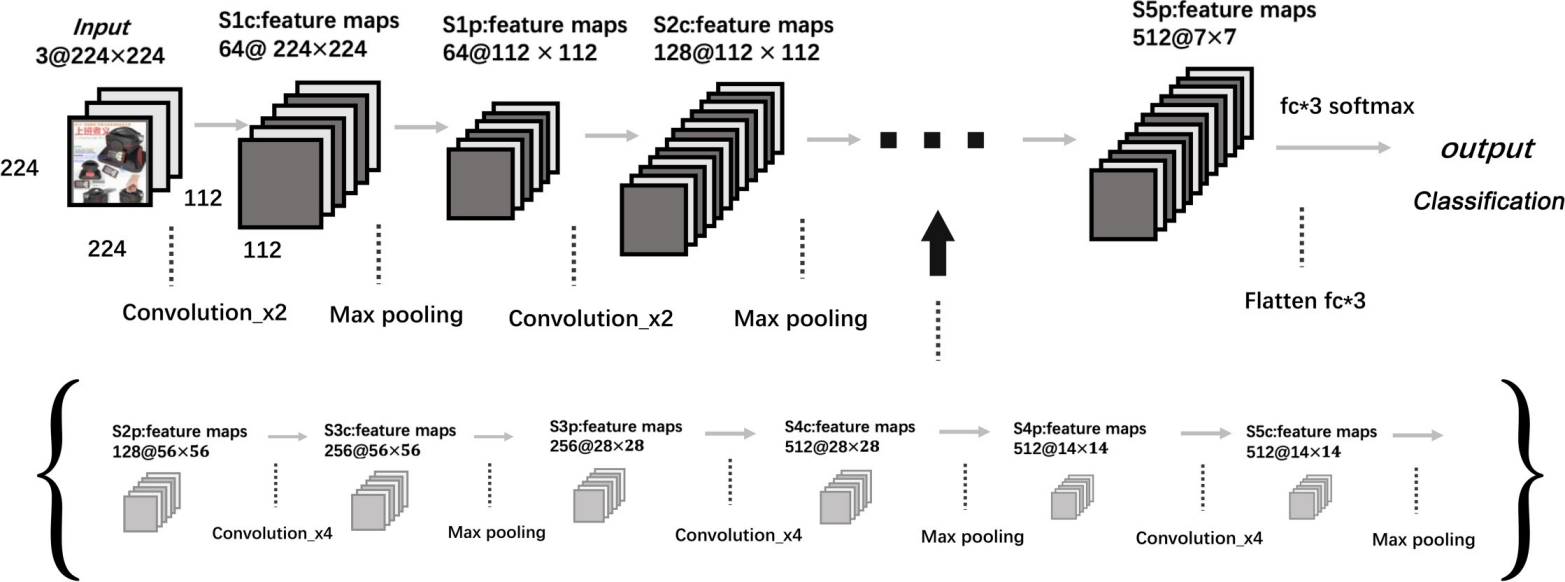
Architecture of the VGG-19 networks. Each plane is a feature map.

Specifically, the soft-max layer is set as the final layer. All hidden layers are using ReLU as the activation function. The VGG-19 network architecture is presented in Fig 1, and the detailed feature extraction process is presented in Table 2.

**Table 2:** Architecture and feature extraction process of VGG-19 for aesthetic-aware modeling.

Layer name	Layers	Output size
Conv1	Conv,3x3,64 × 2,Max pool	[224,224,64],[112,112,64]
Conv2_x	Conv,3x3,128 × 2,Max pool	[112,112,128],[56,56,128]
Conv3_x	Conv,3x3,256 × 4,Max pool	[56,56,256],[28,28,256]
Conv4_x	Conv,3x3,512 × 4,Max pool	[28,28,512],[14,14,512]
Conv5_x	Conv,3x3,512 × 4,Max pool	[14,14,512],[7,7,512]
Dense3_x	Flatten,fc × 3,fc × 3	[25088],[1000],[64]
Output	3d fc, Softmax	[3]

#### ResNet-50

ResNet-50 is applied to extract multimodal features of design work images in view of its feasibility in existing studies [18, 39]. The ResNet-50 training process is shown as follows:

- Firstly, the input image is reset to 224 x 224 before the training process.
- Secondly, we extracted multimodal features by ResNet-50 to form an output vector of 2,048 features. The output vector is further fed into a fully connected network with four fully connected layers.
- Thirdly, the dimensions of image features vector were reduced by the fully connected network for aesthetics classification.

Specifically, various optimized methods were applied in features processing, including Standard Feature Normalization shift, rotation, zoom, nearest-fill and horizontal flip. Then we proceeded to use 2048-dimensional features and the normalized correlation value to train the fully connected network for 300 epochs. In the four-layer fully connected network (FCNN), the activation functions of the other layers are ReLU to prevent the gradient from disappearing or exploding.

Softmax (mapping the result to 0–1) is applied as the activation function in the output layer. The detailed feature extraction process is presented in Table 3.

**Table 3:** Architecture and feature extraction process of ResNet-50 for aesthetic-aware modeling.

Layer name	Layers	Output size
Conv1	Conv,7x7,64.stride 2,Max pool,3x3,stride 2	[112,112,64],[56,56,64]
Conv2_x	$\left[\begin{array}{ccc} Conv & 1\times 1 & 64\\ Conv & 3\times 3 & 64\\ Conv & 1\times 1 & 256 \end{array}\right]\times 3$	[56,56,256]
Conv3_x	$\left[\begin{array}{ccc} Conv & 1\times 1 & 128\\ Conv & 3\times 3 & 128\\ Conv & 1\times 1 & 512 \end{array}\right]\times 4$	[28,28,512]
Conv4_x	$\left[\begin{array}{ccc} Conv & 1\times 1 & 256\\ Conv & 3\times 3 & 256\\ Conv & 1\times 1 & 1024 \end{array}\right]\times 6$	[14,14,1024]
Conv5_x	$\left[\begin{array}{ccc} Conv & 1\times 1 & 512\\ Conv & 3\times 3 & 512\\ Conv & 1\times 1 & 2048 \end{array}\right]\times 3$	[7,7,2048]
Dense3_x	Average Pool,fc×3,Dropout	[512],[128],[64]
Output	3d fc, Softmax	[3]

**Fig 2:**
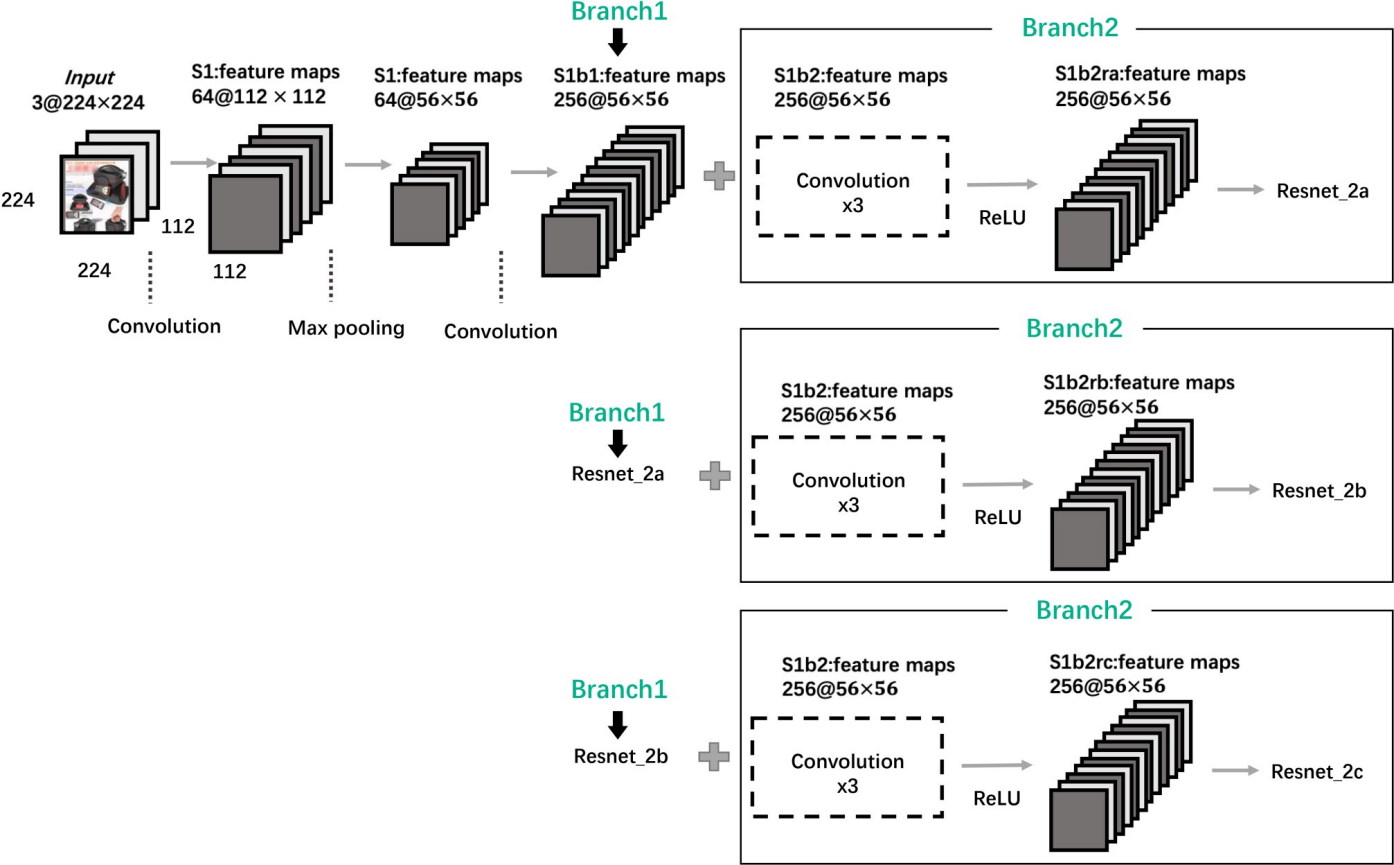
Architecture of the ResNet-50 network. Each plane is a feature map.

As shown in Fig 2, a four-layer fully connected network was introduced to predict similarity after images features training. All activation functions in the first three layers of FCNN are ReLU to prevent gradients from disappearing or gradients exploding, and the activation functions of the output layer is softmax. During the training process, binary cross entropy was set as a loss function and Adam was set as the optimizer.

## Experiments

**Fig 3:**
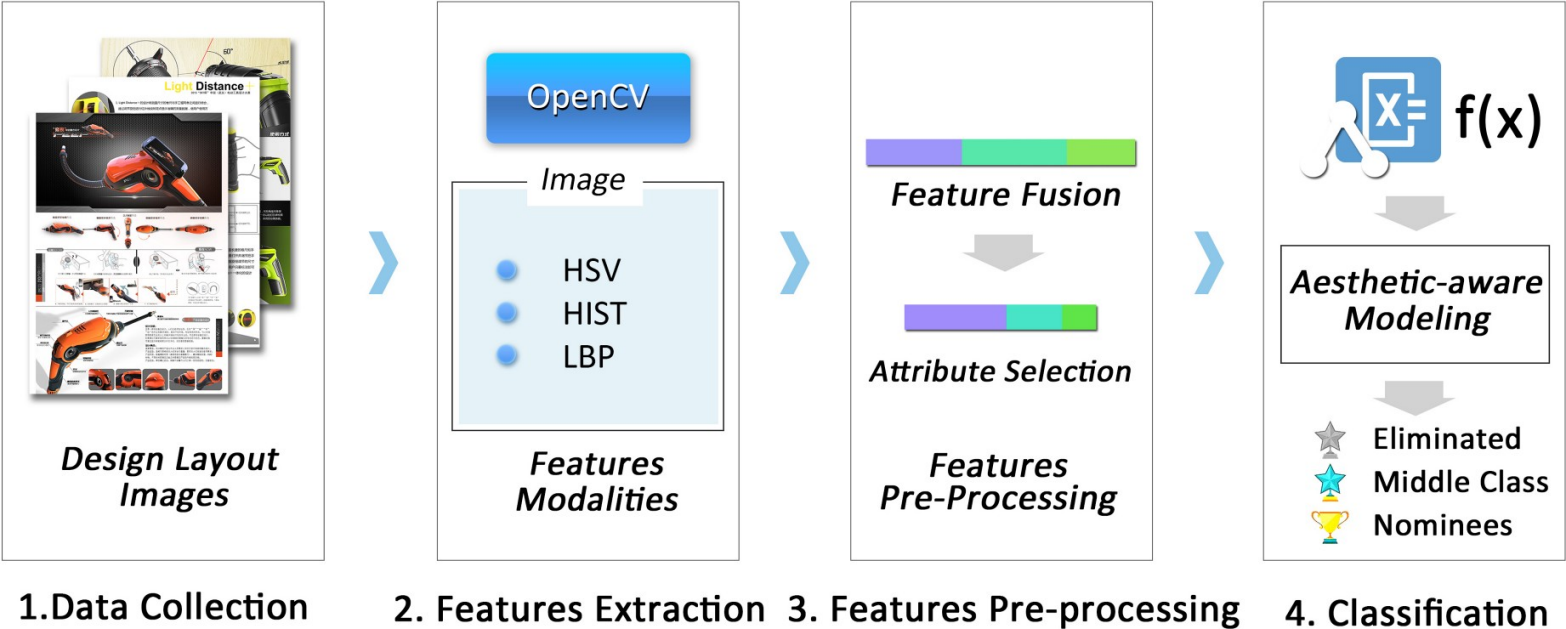
Experimental procedure for aesthetic-aware modeling, based on design awards datasets.

The datasets of applicants for the Electronic Home Applicants Design Awards and the Electronic Tools Design Awards were both divided into three categories: eliminated, middle class, and nominees. The experiments were intended to recognize the design works with low aesthetic scores (eliminated) and with high aesthetic scores (nominees). The competition results appear to indicate that, to some extent, the evaluation of aesthetic features is fundamental to judging design quality. The works of design that receive awards are judged to be outstanding in every aspect. The aesthetic level of a design can thus be a clue in assessing the design quality, transforming this latter task into an aesthetic-level classification question. An general aesthetic-aware modeling experimental procedure is presented in Fig 3.

### 4.1 Reviewers for design awards

Nine experts were reviewers for the 2012 Electronic Home Applicants Design Awards, and five experts were reviewers for the 2015 Electronic Tools Design Awards. These experts are renowned educators and practitioners in industrial design from top universities and design companies in China, and they all have rich experience in evaluating design.

### 4.2 Datasets

Two original datasets were used in this experiment. The first dataset contains images of 2216 design works collected from the 2012 Electronic Home Applicants Design Awards, and the other one contains images of 639 pieces from the 2015 Electronic Tools Design Awards. In the experiment, the Electronic Home Applicants Design Awards database was randomly divided for model exploration, in which 1777 pairs were set for training and 439 pairs were set for testing. While in the Electronic Tools Design Awards database, 519 pairs were randomly selected for training and 129 pairs were selected for testing.

For the aesthetic–aware classification modeling of Liblinear, LibSVM, RBFNetwork, and RSS-Randomforest, image features LBP, HSV, and HIST were used to form the datasets. LBP, HSV, and HIST were extracted in 64, 256, and 256 dimensions, respectively, were extracted using OpenCV. Then, VGG-19 and ResNet-50 were used as a comparison for modeling. We first built the aesthetic model using the Electronic Home Applicants Design Awards dataset, and then we tested it on the dataset from the Electronic Tools Design Award. The detailed feature extraction method is introduced in section 3.2.

### 4.3 Experts’ review procedure for design awards

The steps that the experts followed in their reviews procedure were introduced as follows.

#### Collection of design works

A total of 2247 design works were collected from the Electronic Home Applicants Design Awards in 2012, of which 2216 were usable for image processing. A total of 671 design works were submitted to the Electronic Tool Design Awards in 2015, of which 639 were usable for image processing. The design works were created by college students pursuing an industrial design major and designers working in related industries around the world.

#### Design review

After the submission deadline, a number of experts were invited to rate the design works according to several criteria. The design criteria of the two design awards are listed below: innovation in appearance and function (30%), market value and feasibility (20%), environmental aspect (20%), harmonious color design (10%), layout presentation quality (10%), and comprehensive evaluation (10%) (Table 4). It should be noted that design aesthetic is captured in relation to three items in the review, and it is a crucial element and part of the basic standard for design competitions. Submission scores were obtained for the first round of the experts’ review, with a numerical value on a scale from 0 to 100.

**Table 4:** Evaluation items for Electronic Home Applicants Design Awards and electronic tool design awards.

No.	Items	Description
1	Innovation	Innovative appearance and functions: novelty of the design, novelty of appearance, incorporation of new technology or new materials suitability as part of a new way of life.
2	Feasibility	Market value and feasibility: design concept is suitable for mass production at reasonable cost.
3	Environmental aspect	The design uses material that is not environmentally damaging and conserves energy in its use.
4	Harmonious color design	Harmonious selection and combination of colors in the product design.
5	Layout presentation quality	The design layout conforms to the aesthetic requirements in color and structure.
6	Comprehensive evaluation	An overall impression score, which essentially provides the juries a chance to show their own preference and to encourage those products they consider is interesting and that have potential, in spite of pitfalls for certain aspects.

#### Primary screening of submissions

Works that scored 0–60 were categorized as having poor design quality and were eliminated in this primary round of selection, such that 821 pieces of Electronic Home Applicants Design Awards and 182 pieces of Electronic Tools Design Awards were eliminated in this first round of selection.

#### Nomination

The expert reviewers selected the nominees after the primary selection. These nominees were selected from the submissions that remained after primary screening. A total of 125 pieces were nominated from the pool for the Electronic Home Applicants Design Awards, and 77 pieces were selected as nominees from the pool for the Electronic Tools Design Awards.

#### Final awarding

**Fig 4:**
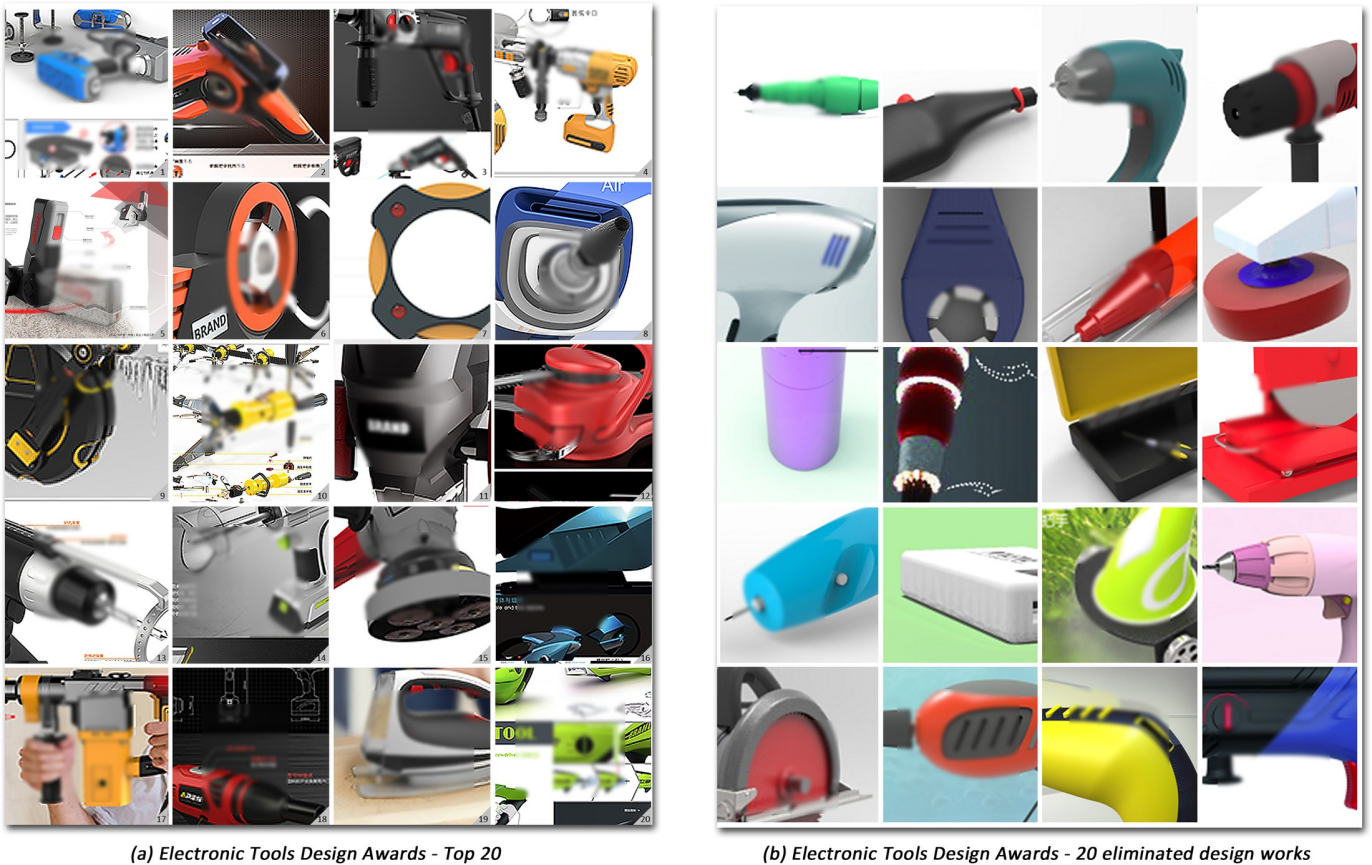
Fig (a) presents layouts of the top 20 design works from the Electronic Tools Design Awards and Fig (b) presents 20 layouts of the eliminated design works in this award as a comparison. Only part of the layout is presented here due to copyright protections.

The awards list was generated by ranking the results for the list of nominees. In this session, the reviewers were invited to thoroughly discuss them and debate the final points. Fig 4 presents layouts of the top 20 design works from the Electronic Tools Design Awards and 20 layouts of the eliminated design works in this award as a comparison.

### 4.4 Aesthetic-aware modeling

Visual aesthetics will influence user preference [7] and acceptance [11] of products. It is relatively difficult to evaluate the creativity level of each work, since it needs a large database of various design concepts, which integrate design factors of text, shape and ergonomics etc. Therefore, we took visual aesthetics character as a research point to carry out evaluation modeling.

In the current aesthetic related research, aesthetic score ranking computing and classification of aesthetic level (positive/negative) are two major modes of aesthetic computing methods. This study proposed an aesthetic classification problem in two design awards datasets. The significance of this paper lies in the utilization of an objective design competition database with ground-truth annotations, which is a suitable carrier of aesthetic computing research.

In this experiment, several algorithms were applied to the datasets to build the model, including LibSVM, Liblinear, RBFNetwork, RandomSubspace-RandomForest, VGG-19 and ResNet-50. Among all these approaches, the learning method of ResNet-50 attained the best accuracy, 74.32% for the dataset of the Electronic Home Applicants Design Award and 73.25% for the dataset of Electronic Tools Design Award. That is, design works were classified more efficiently and with superior accuracy by this approach.

Two experimental sessions were conducted in this study. Firstly, design works that had been scored by experts using design evaluation criteria were evaluated. Secondly, design proposal layouts were evaluated via machine learning. The dataset for the Electronic Home Applicants Design Awards was utilized for modeling exploration, and the method was proved to be effective by the verification using the dataset from the Electronic Tools Design Awards.

## Results and discussion

In this study, we combined multiple features of images for aesthetic evaluation for two design award datasets. A comparison was conducted using LibSVM, Liblinear, RBFNetwork, RandomSubspace-RandomForest, VGG-19 and ResNet-50 to obtain the best model. Using aesthetic-aware model results, ResNet-50 were found to achieve the best classification accuracy.

### Models comparison and optimization

The specific results of the comparison of the algorithms applied to the Electronic Home Applicants Design Awards dataset are presented in Table 5.

**Table 5:** Aesthetic-aware classification accuracy of the dataset from the Electronic Home Applicants Design Awards.

Dataset	Algor.	Results	
		ACC	*Parameters setting*
***Electronic*** ***Home*** ***Applicants*** ***Design*** ***Award***	Liblinear	57.01%	L2-regularized L2-loss support vector (dual), cost 1, eps 0.001
	LibSVM	59.01%	C = 1, Gamma = 0
	RBFNetworks	63.06%	minStdDev 0.1, numClusters 2, ridge 1.0E-8 clusteringSeed = 2
	RSS- Randomforest	59.97%	RSS: subSpaceSize 0.7553, numIterations 9, RF: numIterations 4, numDecimalPlaces 2, maxDepth = 0, seed = 1.
	VGG-19	70.03%	lr = 0.001, training_iters = 1777, batch_size = 128
	**ResNet-50**	**74.32%**	lr = 0.001, training_iters = 1777, batch_size = 128

The performance of modeling could be limited by the scale of dataset, which would ultimately constrain the scalability of the method. Consequently, it was tested in the dataset of the Electronic Tools Design Awards to indicate its effectiveness in aesthetic evaluation. ResNet-50 also attained the best accuracy of 73.25% for the Electronic Tools Design Awards dataset. The verified modeling results of the Electronic Tools Design Awards are shown in Table 6.

**Table 6:** Aesthetic-aware modeling verification in the Electronic Tools Design Awards design award dataset.

Dataset	Algor.	Results	
		ACC	*Parameters setting*
***Electronic*** ***Tools*** ***Design*** ***Award***	Liblinear	59.57%	L2-regularized L2-loss support vector (dual), cost 1, eps 0.001
	LibSVM	59.31%	C = 2, Gamma = 0
	RBFNetworks	61.19%	minStdDev 0.1, numClusters 2, ridge 1.0E-8 clusteringSeed = 1
	RSS- Randomforest	59.31%	RSS:subSpaceSize 0.5, numIterations 10, RF:numIterations 4, numDecimalPlaces 2, maxDepth = 0, seed = 1.
	VGG-19	68.36%	lr = 0.001, training_iters = 519, batch_size = 128
	**ResNet-50**	**73.25%**	lr = 0.001, training_iters = 519, batch_size = 128

The results of model comparisons in Table 5 and Table 6 show that ResNet-50 outperformed other algorithms in the average accuracy of classification.

#### Best features exploration for hand-crafted features

Aesthetic evaluation by hand-crafted features is also an important method in this area. An investigation of best features can provide guidance to the further study. Consequently, the CfsSubset Evaluation via BestFirst method was applied in the feature selection to find the most relevant hand-crafted features with lookupCacheSize of 1 and searchTermination of 5. As a result, 16 relevant image features were selected for “Electronic Home Applicants Design Award” dataset and 26 relevant image features were selected for “Electronic Tools Design Award” dataset. The most relevant features for each dataset are listed in detail in Table 7. In the analysis result, 10 HSV features are most relative to aesthetic character of image for “Electronic Home Applicants Design Award” while 17 HIST features are most relevant for Electronic Tools Design Award dataset, see Table 7. The results indicate that the best features for aesthetic recognition can be differ in different datasets. It might be due to the difference of image content. It can be concluded that color related features should be concerned in design evaluation. Accordingly, HSV and HIST can be endowed with more concern in the further study.

**Table 7:** Best features selection by CfsSubsetEvaluation via BestFirst method.

Dataset	Type	Numbers of features
***Electronic*** ***Home*** ***Applicants*** ***Design Award*** ***(16)***	HSV	10
	HIST	1
	LBP	5
***Electronic*** ***Tools*** ***Design Award*** ***(26)***	HSV	8
	HIST	17
	LBP	1

#### Classification performance analysis

The classification results indicate that a relatively higher classification effect was found for the class of nominees than the classes of the eliminated, see Table 8. The recognition of awarded design works was found to be relatively effective in the model exploration. The experimental result proved that the aesthetic level can be a cue for general design quality assessment. This may be because good presentation design is considered as a basic requirement of a design concept submission. Consequently, those design works that have a poor appearance are eliminated in the first round. Likewise, design works of higher quality share common design characteristics, such as detailed product images and descriptive text, along with visual and attractive color schemes. Nevertheless, when it comes to the final round of the selection for the design award, it is difficult to isolate the best works using layout and appearance alone, so the final ranking of the nominees might be hard to predict by aesthetic modeling. In the further study of intelligent design evaluation, semantic analysis should be incorporated to comprehend the highlights of design thinking.

**Table 8:** Aesthetic-aware classification accuracy comparison using VGG-19 and ResNet-50.

Dataset	Class	*Detailed accuracy by class*	
		VGG-19	ResNet-50
***Electronic*** ***Home*** ***Applicants*** ***Design*** ***Award***	Eliminated	61.78%	66.67%
	Nominees	93.33%	93.70%
***Electronic*** ***Tools*** ***Design*** ***Award***	Eliminated	67.03%	75.95%
	Nominees	83.59%	84.19%

**Fig 5:**
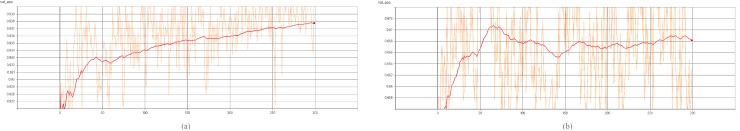
Loss during ResNet-50 training process for Electronic Home Applicants Design Award dataset. (Fig (a):Nominees classification accuracy; Fig(b): Eliminated classification accuracy).

**Fig 6:**
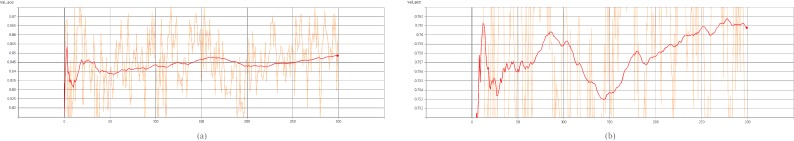
Loss during ResNet-50 training process for Electronic Tools Design Award dataset. (Fig (a): Nominees classification accuracy; Fig (b): Eliminated classification accuracy).

We proceeded to explore the two-class image classification to distinguish the eliminated and un-eliminated ones, and classified the nominees from all the submissions by deep learning methods. In the ResNet-50 training process, the optimal classification accuracy achieves stable after 300 epochs of training, see Fig 5 and Fig 6. For the Electronic Home Applicants Design Award database, the Nominees classification accuracy achieves 93.70% after 300 epochs of training using ResNet-50, and the Eliminated classification accuracy achieves 66.67%. For the Electronic Tools Design Award database, the Nominees classification accuracy achieves 84.19% after 300 epochs of training using ResNet-50, and the Eliminated classification accuracy achieves 75.95%.As a result, the average classification accuracy of ResNet-50 outperforms the performance of VGG-19, see Table 8.

It is interesting to consider that if a design achieves low score in presentation alone, it could be considered not to qualify for consideration for an award. The principle that appearances matter holds true as well for artificial intelligence aesthetics perception. All the design works that received awards also conformed to high standards in the design of their presentation posters. Our experimental results confirmed our hypothesis that aesthetics can be assessed using a machine learning approach, and features that fuse the modeling of design layout images may be a feasible avenue for development of intelligent aesthetic perception.

## Conclusion and directions for future work

Although the style of human cognition used in art and design is abstract and subjective, the scientific exploration of feature dimensions and data fusion can allow computers to obtain a sense of appreciation for design. In design work, layout follows certain formatting and color-combination rules. Comparing to paintings and abstract works of art, containing much personal understanding and preference, the aesthetic patterns of design layout can be studied using machine learning methods more readily.

In this study, we created an original database for the aesthetic evaluation of art and design, which may become a useful data resource for multimedia aesthetic computing. We also created an effective method for the aesthetic evaluation of design layouts based on multi-modal image features. In this work, 2,981 original design works taken from entrants of two design competitions were collected to build two sets of data for the design of aesthetic images. One dataset was used for the construction of models, and the other was used for aesthetic-aware model testing. In our experiment, the image features LBP, HIST, and HSV were extracted to form the dataset for traditional machine learning approaches. Subsequently, VGG-19 and ResNet-50 were used for comparison with the results of traditional method. The best aesthetic evaluation result reached a classification accuracy of around 80% for both datasets based on ResNet-50, and the classification was more accurate for the nominated designs than the eliminated ones. The experimental findings suggest that aesthetic-aware modeling based on image feature analysis is a feasible approach for automatic design evaluation. Software can acquire the ability of aesthetic appreciation by following this promising methodology.

Many possible avenues exist for future work. A larger dataset of design works should be built to improve modeling accuracy. Thus, it would be possible to use fusion deep learning methods for feature extraction and classification for model optimization. To verify this method, a system of design evaluation can be developed, based on the model explored for design competition review or self-assessment in designing. Moreover, this method can be used in possible applications in related areas of packaging and advertisement design involving image aesthetics assessment. Subsequent this study can involve the use of the method to address questions of aesthetic perception in various scenarios.

## Supporting information

S1 File Design aesthetic database description. (DOCX)
