# Supplementary material for: And the nominees are: Using design-awards datasets to build computational aesthetic evaluation model
Source: PLoS One. 2020 Jan 21;15(1):e0227754. doi: 10.1371/journal.pone.0227754 (PMC6974033; doi:10.1371/journal.pone.0227754)
Supplement: S1 File — (DOCX) [file pone.0227754.s001.docx]

**Design Aesthetic Database Description**

The Design Aesthetic Database includes two types of datasets:

**1. Handcrafted features datasets:**

Electronic Home Applicants Design Award-handcrafted features.csv

Electric Tools Design Award-handcrafted features.csv

Electronic Home Applicants Design Award-handcrafted features.csv and Electric Tools Design Award-handcrafted features.csv are the datasets of handcrafted features extracted from the design layout images. Local Binary Pattern (64 dimensions), Color Histogram (256 dimensions), and Hue Saturation Value (256 dimensions) features were extracted to form the datasets. A total of 576 dimensions of image features were extracted to form each dataset.

**2. Features extracted by RESNET-50:**

Electronic Home Applicants Design Award-RESNET.csv

Electric Tools Design Award-RESNET.csv

Electronic Home Applicants Design Award-RESNET.csv and Electric Tools Design Award-RESNET.csv are the datasets of features extracted by RESNET-50. A total of 25,088 dimensions of image features were extracted to form each dataset.

**Design Aesthetic Database** can be found via:

https://figshare.com/s/b5fd6afaf0af7e1cf3b0
